# The Mediating Role of Perceived Stress and Academic Procrastination between Physical Activity and Depressive Symptoms among Chinese College Students during the COVID-19 Pandemic

**DOI:** 10.3390/ijerph20010773

**Published:** 2022-12-31

**Authors:** Leshui Yang, Zongyu Liu, Shengnan Shi, Ye Dong, Huijun Cheng, Tuojian Li

**Affiliations:** School of Physical Education, Shandong University, Jinan 250061, China

**Keywords:** physical activity, depression, perceived stress, academic procrastination, college students, cross-sectional study, mediation analysis, mental health, depressive symptoms

## Abstract

Depressive symptoms, a prevalent mood illness, significantly harm college students’ physical and mental health. Individuals have experienced some degree of psychological harm as a result of the COVID-19 pandemic. Taking this into account, the purpose of this study was to investigate the relationship between physical activity (PA) and depressive symptoms among college students during the COVID-19 pandemic, as well as the mediating roles of perceived stress and academic procrastination. A total of 586 college students were subjected to the Physical Activity Scale (PARS-3), the Perceived Stress Scale (PSS-10), the Procrastination Assessment Scale-Students (PASS), and the Patient Health Questionnaire (PHQ-9). Findings from this research demonstrated that there was a significant positive correlation between perceived stress, academic procrastination, and depressive symptoms, while PA was significantly negatively correlated with perceived stress, academic procrastination, and depressive symptoms. The results of the chain mediation analysis showed that PA had a significant direct effect on depressive symptoms. Perceived stress, academic procrastination, and perceived stress-academic procrastination had significant mediating and chain mediating effects on the relationship between PA and depressive symptoms. In conclusion, PA among college students during the COVID-19 pandemic affects their depressive symptoms directly and indirectly through the independent mediating effect of perceived stress and academic procrastination, as well as the chain mediating effect of perceived stress and academic procrastination.

## 1. Introduction

As the COVID-19 widespread disease continues to wildly attack countries, it has disrupted academic, social and future planning for college students and negatively affected their mental health [1,2]. Depressive symptoms, as a common mood disorder, have become one of the main mental health issues in the world [3]. College students are a special group who are full of energy but immature in mental development [4]. When college students are in such a state of depressed mood, they often feel extremely restless and have a low mood long-term [5]. In terms of actions, behaviors such as self-harm or even committing or attempting suicide may occur [6]. The investigation showed that the detection rate of depression among Chinese college students was generally more than 20%, and the level of depression among them was gradually rising over the increase of years [7]. Depression is common among college students, and some studies believe that the depression of college students is more serious than that of their non-college peers [8]. College students’ study performance and quality of life are directly impacted by depression [9]. Theoretically and practically, it is important to investigate the causes and mechanisms of depression in college students.

### 1.1. The Role of PA on Depressive Symptoms

Many cross-sectional studies have shown that there is a negative correlation between PA and depression in college students, which means that the greater the amount of PA, the lower the score or detection rate of depression [10,11,12]. By examining correlations between baseline PA levels among college students and depression detection rates or scores at follow-up, other longitudinal studies have discovered comparable findings [13,14]. Additionally, a meta-analysis study [15] showed that exercise has a positive antidepressant impact. PA has gained a lot of attention in the past decade as a treatment for depression, which can be an alternative approach that individuals can use to cope with depression, either on their own or helped by others. A study from the US reported that participants who reduced PA before and after COVID-19 increased their likelihood of suffering depression [16]. There is also evidence that physical exercise alleviates depressive symptoms among female college students who suffer from mild and moderate depression [17]. Feter et al. found that those who started or maintained physical activity in the first three months before the COVID-19 pandemic were at lower risk of exacerbating depressive symptoms than those who were inactive and that this relationship was stronger during the pandemic [18]. In order to offer the theoretical foundation for reducing college students’ depressive symptoms and boosting their mental health, this study intends to examine the underlying mechanism of PA influencing college students’ depressive symptoms during COVID-19.

### 1.2. The Mediating Role of Perceived Stress between Physical Activity and Depressive Symptoms

Recent research indicates that the association between PA and depression may be influenced by perceived stress and academic procrastination. The degree to which a person believes their life is unpredictable, out of their control or overcrowded is referred to as perceived stress [19]. Improving overall health and function is the primary purpose of PA [20]. Moderate PA has a cognitive and behavioral activation effect on individuals, supports psychological equilibrium, and increases good feelings and self-awareness [21]. Physical exercise has the power to relieve psychological stress in addition to strengthening the body and preventing disease [22]. College students are at a more vulnerable stage [23], and the strain from their studies and other obligations is growing. This pressure directly contributes to interpersonal conflict and gives rise to negative feelings like jealousy, dissatisfaction, and animosity. However, this condition can be lessened by PA. Crew et al. pointed out that those who regularly exercise have fewer physiological stress reactions than those who are sedentary [24]. According to research by Unger et al., moderate-intensity PA can help the body better handle psychological stress by reducing the negative effects of acute and chronic psychological stress on its psycho-neuro-immune function [25]. Qi et al. revealed that PA was negatively associated with perceived stress despite the fact that participants’ PA levels decreased with the continued spread of COVID-19 [26]. This suggests that the negative association between PA and perceived stress is supported by evidence both before and after the COVID-19 outbreak. Studies on the connection between perceived stress and depression have produced similar results. For instance, a study revealed that adolescents’ perceived stress caused by the COVID-19 pandemic was significantly and favorably correlated with depressive symptoms [27]. According to the cognitive vulnerability-stress theories of depression, stress and a vulnerable cognitive style work together to facilitate the emergence and progression of depression [28]. High levels of perceived stress have been linked to aberrant cortisol secretion and dysfunctions of the hypothalamic-pituitary-adrenal cortex (HPA) axis, which can result in physical and mental illnesses such as insomnia, cardiovascular disease, obesity, and depression [29]. This shows that by affecting how stressed out a person feels, PA may affect their chance of developing depression.

### 1.3. The Mediating Role of Academic Procrastination between PA and Depressive Symptoms

Procrastination has been found to be an instinctive response to anxiety, stress, etc [30]. College students must contend with the stress of academic rivalry, difficult interpersonal relationships, and other issues as they progress through an important stage in their personal development. According to the theory of planned behavior, the disconnect between intention and action causes academic procrastination [31]. Behavioral attitude, subjective norms, and perceptual behavioral control all influence each person’s behavior intention. Individuals may face physical and psychological challenges in the process of physical training. The negative association between PA and procrastination was confirmed both before and after the pandemic outbreak. Li et al. discovered that a PA intervention aiming at enhancing self-control and self-efficacy helped lessen college students’ academic procrastination [32]. PA can thus help people create positive behavioral intentions and reduce or eliminate academic procrastination. A cross-sectional study conducted by Shi et al. during the COVID-19 pandemic also found that PA had a positive effect on reducing procrastination and that higher-intensity PA had a greater effect on irrational procrastination [33]. Academic procrastination is the behavior or behavior tendency of students to put off or delay finishing academic tasks in learning activities or learning situations [34]. Long-term academic procrastination may have a negative impact on health [35], as well as a decline in academic performance for college students. Additionally, studies have shown that procrastinating is a negative and maladaptive activity that is frequently associated with unpleasant emotional states, including worry and despair [36]. Beutel et al. found a positive association between academic procrastination and perceived stress and depression [37]. Peixoto et al.’s study during the COVID-19 pandemic similarly yielded a positive relationship between academic procrastination and individual adverse psychological moods (including depression) [38]. Due to this, we posit that PA may reduce depressive symptoms by reducing procrastination.

### 1.4. The Current Study

**Figure 1:**
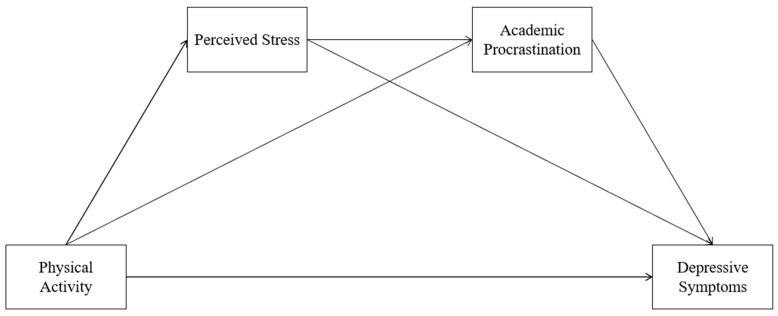
Hypothesized relationships between PA, perceived stress, academic procrastination and depressive symptoms.

College students’ academic performance, physical health, and mental health are all greatly impacted by academic procrastination and depression, which is not good for their future development. Therefore, strategies must be developed to lessen the epidemic’s level of academic procrastination and sadness. This study aims to investigate the association between PA and depressive symptoms in college students as well as the impact of perceived stress and academic procrastination based on the aforementioned literature. The study proposed that perceived stress and academic procrastination served as a chain mediating role between college students’ PA and depression and that there was a strong negative association between college students’ PA and depressive symptoms. We developed a theoretical hypothesis model (shown in Figure 1) based on prior empirical research and put forth the following four hypotheses: (1) PA can positively predict the depressive symptoms of Chinese college students. (2) PA can indirectly predict depressive symptoms of college students through the mediating role of perceived stress. (3) PA can indirectly predict depressive symptoms of college students through the mediating role of academic procrastination. (4) PA can indirectly predict depressive symptoms of college students through the chain mediation role of perceived stress and academic procrastination.

## 2. Materials and Methods

### 2.1. Participants and Procedures

The study was designed as a cross-sectional survey, and we covered the sample in various regions of China. The sample size was calculated based on the suggested ratio of ten participants per observable variable. Thus, given that the scale consists of 34 items, a sample size of at least 340 valid cases is required. However, due to the outbreak of the COVID-19 pandemic and the closure of some universities, offline surveys were difficult. Therefore, based on the advice of scholars, we used a snowball sampling method to recruit current university students to complete the online survey [39]. The study was conducted in May and June 2022. We first contacted the authors’ former classmates who were graduate students at the university, and asked them to invite undergraduate students from their schools to participate in this survey. We asked them to send the link to the electronic questionnaire to the participants. To increase the response rate of the questionnaire, we sent a cover letter containing detailed information about the purpose of the study, data confidentiality assurance, and personal anonymity of the participants before conducting the formal survey. We have also clarified that there were no right and wrong answers to any questions and that their responses were valuable to us. Students from eight universities in Shandong, Sichuan, Hainan, and Shaanxi provinces took part in the survey. A total of 620 questionnaires were sent (155 questionnaires were sent out in each region), and 586 valid questionnaires were collected, with an effective rate of 94.52%. There were 334 boys and 252 girls, with an average age of 20.26 years and a standard deviation of 1.78 years. Demographic characteristics were collected through several sociodemographic questions, including age, sex, urban-rural provenance, whether the family is a single parent, self-assessment of academic performance, daily sleep hours, etc.

### 2.2. Measures

#### 2.2.1. Physical Activity Rating Scale (PARS-3)

Liang et al. [40] redesigned the physical Activity Rating Scale (PARS-3) and used it to assess individuals’ recent one-month PA levels. The three items on the scale—intensity, duration, and frequency of physical activity—are used to quantify the quantity of exercise. The intensity and frequency of exercise are divided into 1 to 5 grades, with 1 to 5 points, respectively. Exercise time is divided into 1~5 grades and 0~4 points, respectively. The scoring method is the amount of exercise = intensity × time × frequency, the highest score is 100 points, and the lowest is 0 points. The assessment criteria for physical activity are: a low physical activity rate of ≤19 points, a moderate physical activity rate of 20–42 points, and a high physical activity rate of ≥43 points. The Cronbach’s α coefficient of the scale in this study was 0.714.

#### 2.2.2. Perceived Stress Scale (PSS-10)

The Perceived Stress Scale (PSS-10) was used in this study to assess the perceived stress levels in participants’ lives [41]. This version had ten items indicating how often participants found their lives unpredictable or overloaded in the last month, classified on a Likert scale from 0 (never) to 4 (very often). Questions 4, 5, 7, and 8 have a reverse score. Final scores ranged from 0 to 40, with higher scores indicating higher levels of perceived stress among participants. The Cronbach’s α coefficient of the scale in this study was 0.781.

#### 2.2.3. Procrastination Assessment Scale—Students (PASS)

Academic procrastination was measured by the academic procrastination Scale [42] developed by Solomon and Rothblum in 1984. The scale adopts a 5-level scoring method, and the item score ranges from 1 to 5 points from low to high. This study adopts the first part of the scale, which consists of six academic dimensions, and each dimension contains three questions. The first two questions are used to measure the level of academic procrastination of college students, and the third question is about the value of college students’ expectations to reduce academic procrastination. In the current study, we used the first two items in each domain. The sum of the PASS items delivers an overall measure of academic procrastination, with the total score ranging from 12 to 60 points. The higher a student’s score is, the more serious his academic procrastination is. The Cronbach’s α coefficient of the scale in this study was 0.869.

#### 2.2.4. Patient Health Questionnaire (PHQ-9)

The Patient Health Questionnaire [43] was used to evaluate the depressive symptoms of the subjects in the last two weeks. This scale was suitable for the general population. For example, it was validated for Chinese university students and the citizen population [44,45]. There were nine items in total, and the score was 0 (none at all)–3 (almost every day). The total score ≥ 5 indicated that the subjects had depressive symptoms. The higher the score, the more severe the depressive symptoms. The Cronbach’s α coefficient of the scale in this study was 0.905.

### 2.3. Statistical Analysis

SPSS 22.0 software was used for analysis. We first conducted descriptive statistics for the four variables and then standardized the data of these variables. Since our data may not be normally distributed for all variables due to the small sample size, we used Spearman correlation analysis. Bootstrapping was used in the mediation analysis because it is a nonparametric resampling technique involving random and repeated subsampling of the data, with no need to satify the assumption of normally distributed data [46]. To test the study hypothesis, we conducted the spearman correlation analysis to explore the relationship between PA, perceived stress, academic procrastination, and depressive symptoms. We then used the SPSS Process Macro 3.3 software to investigate the mediating role of perceived stress and academic procrastination between PA and depressive symptoms, which was specifically developed to test complex models. In this process, Model 6 is applied to the two mediation variables. Indirect effects were calculated using a bootstrap for bias correction. If the 95% confidence interval (CI) does not include 0, it means that the mediation effect is significant. Demographic variables were used as covariates of the model.

## 3. Results

### 3.1. Common Method Deviation Test

Unrotated exploratory factor analysis was performed for all measurement items using the Harman single-factor test. The results showed that a total of seven common factors with eigenvalues greater than one were proposed, and the first common factor explained 28.83% of the total variation, which was less than the 40% judgment criterion. It can be considered that there is no obvious common method bias in this study.

### 3.2. Differences in Perceived Stress, Academic Procrastinationz and Depressive Symptoms among College Students with Different Levels of PA

To further explore the differences in perceived stress, academic procrastination, and depressive symptoms among college students with different amounts of PA, PA was classified into three levels based on previous studies: low PA (*n* = 296), moderate PA (*n* = 139), and high PA (*n* = 151). The results of variance (ANOVA) analysis (Table 1) showed that college students with different levels of PA have significant differences in their perceived stress, academic procrastination and depressive symptoms. In terms of perceived stress, the high PA group was significantly lower than the low and moderate PA groups (F = 13.166, *p* < 0.001). In terms of academic procrastination, the high PA group was significantly lower than the low PA group and the moderate PA group (F = 17.035, *p* < 0.001). In terms of depressive symptoms, the high PA group was significantly lower than the low and moderate PA groups (F = 40.144, *p* < 0.001).

### 3.3. Descriptive Statistics and Correlation Analysis

The correlation coefficients and descriptive statistical results among variables are shown in Table 2. Correlation analysis showed that perceived stress, academic procrastination, and depressive symptoms were positively correlated, while PA was negatively correlated with perceived stress, academic procrastination, and depressive symptoms.

### 3.4. Mediation Analysis

**Figure 2:**
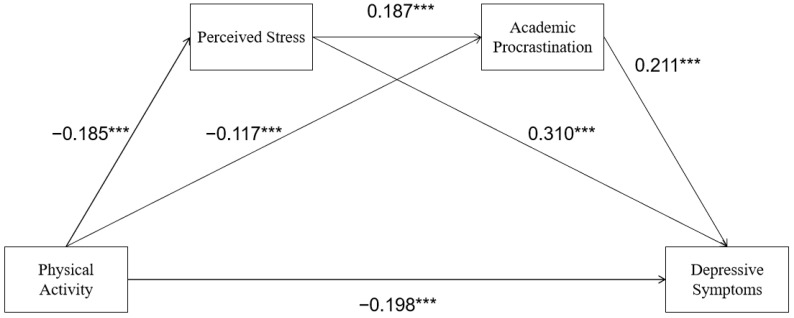
The chain mediating effect of perceived stress and depressive symptoms. *** *p* < 0.001.

The variables in the study were standardized, with PA as the independent variable, depressive symptoms as the dependent variable, and perceived stress and academic procrastination as the mediating variables. Model 6 of the PROCESS plug-in in SPSS was selected to test the independent mediating effect and chain mediating effect. The results are shown in Table 3 and Figure 2. The results of regression analysis showed that PA negatively affected perceived stress, academic procrastination, and depressive symptoms. Perceived stress can positively influence academic procrastination and depressive symptoms. Academic procrastination was positively correlated with depressive symptoms.

The results of the Bootstrap analysis (5000 samples) (Table 4) showed that PA had a significant direct predictive effect on depressive symptoms, and the independent mediating effect of perceived stress and academic procrastination and the chain mediating effect of perceived stress-academic procrastination were all significant. The total indirect effect was 31.4%, the mediating effect of perceived stress was 20.2%, the mediating effect of perceived stress was 8.7%, and the chain mediating effect of perceived stress and perceived academic procrastination was 2.4%.

## 4. Discussion

The results of this study indicated that PA could not only directly affect the depressive symptoms of college students but also indirectly affect depressive symptoms through the mediating effect of perceived stress and psychological procrastination. The mediating effect was realized through three paths: the independent mediating effect of perceived stress, the independent mediating effect of academic procrastination, and the chain mediating effect of perceived stress and academic procrastination.

This study found that the depression rate of Chinese college students during COVID-19 was 48.0%, higher than the 36.56% result, which was found in Yang et al.’s study [47]. This may be due to the large scope and high infectivity of COVID-19, as well as the closed management environment adopted by the school, leading to a certain fear of COVID-19 among college students and affecting their depressive symptoms. Additionally, our study validated earlier research by confirming a substantial negative link between college students’ level of PA and depressive symptoms during COVID-19. Exercise improves students’ physical fitness, calms anxious psychological feelings, and aids in the prevention and treatment of mental illnesses, including depressive symptoms and anxiety, according to earlier research [10,13,14,48,49]. College students’ involvement in PA during the pandemic can have a variety of effects on the severity of depressive symptoms [18]. College students are under considerable pressure due to the epidemic, but they can relieve some of their bad feelings to some extent by exercising [50]. Additionally, regular engagement in PA might raise students’ subjective levels of support and problem-solving skills, lessen distressing emotional experiences, and ultimately lessen depressive symptoms [51]. Moreover, PA can also improve an individual’s depression levels by altering the composition of the gut microbiota, which further affects the secretion of a range of substances that regulate neural activity, such as serotonin [52].

A prior investigation, however, revealed no connection between PA and depressive symptoms [53]. This may be due to the fact that PA’s effect on depressive symptoms is neither specific nor direct, indicating that there may be a mediator between the two elements. The findings of this study imply that perceived stress and academic procrastination may be significant mediators of the COVID-19 epidemic. As a result, it is challenging to predict changes in depression levels directly from changes in PA levels. According to our research, the relationship between PA and depressive symptoms is mediated by perceived stress, which accounts for 18.9% of the overall effect. College students’ levels of depressive symptoms significantly correlate with perceived stress [19,54], a risk factor for mental health. College is an important stage of individual development, and individuals may experience certain changes both physically and mentally, especially in the context of the current COVID-19 pandemic [55]. College students frequently encounter high levels of stress owing to the effect of their surroundings and changes in hormone levels, which may cause emotional difficulties and raise the risk of depression even when the person’s cognitive ability is progressively maturing at this point [56]. College students are constantly subject to the emotional pressure and suffering of others, which unquestionably has a detrimental effect on their mental health. Exercise increases endorphin production, which improves mood and leads to physiological adaptations in blood pressure, resting heart rate, and stroke volume [26]. Regular PA enhances cardiovascular, musculoskeletal, and overall performance, which helps to lower perceived stress [57,58]. Additionally, COVID-19-related quarantine procedures could increase physical inactivity and stress levels [59]. However, a high level of perceived stress can easily cause an individual to worry excessively [60], which leads to a cognitive evaluation of reality that deviates from reality and causes anxiety, despair, and other bad feelings. Therefore, it is necessary to increase publicity and correct guidance to promote individuals or groups to take effective countermeasures.

The mediating effect test also showed that PA not only directly predicted the risk of depression but also indirectly predicted the risk of depression through the mediating effect of academic procrastination. According to some researchers, procrastination results from a failure of individual self-regulation, but PA can improve individual autonomous functions from the standpoint of time management [61]. College students’ levels of PA are decreased as a result of the epidemic’s constraints, which has an adverse effect on their capacity for time management and encourages procrastination. Exercise can help people finish activities in a favorable state and reduce procrastination behavior from a physiological perspective by relieving brain tiredness and enhancing the response and flexibility of brain nerves [62]. Procrastination and depressive symptoms have been connected, according to the findings of a prior study [63]. Another recent study in Kashan discovered a strong correlation between college students’ academic procrastination and depressive symptoms [64]. Valenzuela et al. noted that academic procrastination increases stress and results in the accumulation of academic tasks, which can be a cause or effect of depressive symptoms [65]. Academic accomplishment in college is intimately tied to future development, and procrastinating on assignments can have a detrimental psychological impact on many facets of personal and social life (such as anxiety and depressive symptoms). The association between academic procrastination and perceived stress on the relationship between PA and depressive symptoms in college students during COVID-19 was further illuminated by the chain mediating effect analysis. Regular PA is crucial for treating and preventing chronic diseases, and it has a number of positive effects on mental health [66]. Academic procrastination can have adverse repercussions if it is severe or regular. Stress during the outbreak may cause college students to experience negative emotions and behave poorly. According to the study, people who experience stress are more likely to use procrastination as a coping mechanism, which might result in depressive symptoms among college students [30,34,36,67].

This study is significant because it is the first to look at how perceived stress and academic procrastination affect the relationship between PA and depressive symptoms in college students. PA directly and indirectly affects depressive symptoms in college students through perceived stress and academic procrastination. This study also reveals the psychological mechanisms underlying the relationship between PA and depressive symptoms in Chinese college students during COVID-19, and it offers a theoretical framework on which the government and educational institutions can base specific policy decisions.

However, this study also has certain limitations. First, future research on prospective design is required because the cross-sectional design cannot infer a causal relationship. Second, the complete closure of some universities and the shift to online learning due to outbreak control measures increased the difficulty of collecting a sample. Therefore, the sample size of this study is relatively small, and future studies with large samples of university student populations are needed. Third, the research objects of this study are Chinese college students, and future studies on Chinese adolescents, including representative samples of the whole country, should be carried out. Moreover, when gathering data, the self-report approach is vulnerable to information bias. Last but not least, additional research is needed to determine whether the association between PA and depressive symptoms is likewise mediated or moderated by other factors.

## 5. Conclusions

In conclusion, PA during the COVID-19 epidemic had a direct impact on college students’ depressive symptoms. Additionally, PA indirectly affects depressive symptoms among college students through the chain mediating impact of perceived stress and academic procrastination. Researchers, educators, and mental health workers need to focus on the perceived stress and academic procrastination of college students, as well as intervene in their depressive symptoms. Colleges and universities should make a reasonable effort to schedule enough physical education classes, host a variety of sporting events, and motivate students to participate actively in sports and get daily exercise. Moreover, it is necessary to reduce the perceived stress level of college students during the epidemic and to use educational programs as much as possible to guide college students to reduce academic procrastination.

## Figures and Tables

**Table 1:** Differences in perceived stress, academic procrastination and depressive symptoms of college students with different levels of PA (*n* = 586).

Variable	Low PA	Moderate PA	High PA	F
Perceived Stress	29.17 ± 3.73	28.14 ± 4.16	26.77 ± 4.82	13.166 ***
Academic Procrastination	30.76 ± 7.31	28.24 ± 9.06	26.85 ± 8.20	17.035 ***
Depressive symptoms	8.07 ± 5.41	4.58 ± 6.25	3.52 ± 5.18	40.144 ***

Note: *** *p* < 0.001.

**Table 2:** Descriptive statistics and correlation analysis results (*n* = 586).

Variables	1	2	3	4
1 Physical Activity	1.000	-	-	-
2 Perceived Stress	−0.244 ***	1.000	-	-
3 Academic Procrastination	−0.216 ***	0.215 ***	1.000	-
4 Depressive symptoms	−0.417 ***	0.409 ***	0.325 ***	1.000
M	27.50	28.31	29.16	6.07
SD	25.70	4.25	8.15	7.75

Note: *** *p* < 0.001.

**Table 3:** Regression analysis of the mediating model between perceived stress and academic burnout and physical activity and depressive symptoms.

Variables	Perceived Stress			Academic Procrastination			Depressive Symptoms		
	β	SE	*t*	β	SE	*t*	β	SE	*t*
Physical Activity	−0.185	0.042	−4.407 ***	−0.117	0.041	−2.820 ***	−0.198	0.039	−5.126 ***
Perceived Stress				0.187	0.040	4.645 ***	0.310	0.038	8.201 ***
Academic Procrastination							0.211	0.038	5.525 ***
*R* ^2^	0.256			0.346			0.505		
F	8.118			13.128			28.315		

Note: *** *p* < 0.001.

**Table 4:** Analysis of the mediating effect of perceived stress and academic procrastination on the relationship between physical activity and depressive symptoms and its effect size.

Effect Types	Path	95% CI	Effect	Effect Size
Direct effect	Physical Activity → Depressive symptoms	(−0.273, −0.122)	−0.198	68.6%
Indirect effect	Physical Activity → Perceived Stress → Depressive symptoms	(−0.086, −0.032)	−0.058	20.2%
	Physical Activity → Academic Procrastination → Depressive symptoms	(−0.047, −0.006)	−0.025	8.7%
	Physical Activity → Perceived Stress → Academic Procrastination → Depressive symptoms	(−0.014, −0.003)	−0.007	2.4%
Total indirect effect		(−0.126, −0.057)	−0.090	31.4%
Total effect		(−0.369, −0.206)	−0.287	

## Data Availability

The data presented are available on request from the corresponding author.
